# Good’s Syndrome Mirrors a Combined Immunodeficiency with Anti-Cytokine Antibodies in the Total Absence of B Cells

**DOI:** 10.1007/s10875-026-01992-5

**Published:** 2026-02-17

**Authors:** Aunonna Kabir, Louise Gilbert, Dornaz Almasizadeh, Reza Alizadehfar, Vanessa Polito, David Langlais, René P. Michel, Christos M. Tsoukas

**Affiliations:** 1 Department of Experimental Medicine, McGill University, Montreal, QC Canada; 2 Research Institute of the McGill University Health Centre, McGill University, Montreal, QC Canada; 3 Department of Microbiology and Immunology, McGill University, Montreal, QC Canada; 4 Department of Medicine, Division of Allergy and Clinical Immunology, McGill University Health Centre (MUHC), 1001 Decarie Boulevard, Montreal, QC H4A 3J1 Canada; 5 Dahdaleh Institute of Genomic Medicine, McGill University, Montreal, QC Canada; 6 Department of Human Genetics, McGill University, Montreal, QC Canada; 7 Department of Pathology, McGill University, Quebec, Canada

**Keywords:** Good’s syndrome, Immunodeficiency, B-cells, Anti-cytokine antibodies, Thymoma

## Abstract

Good’s Syndrome, an adult-onset immune deficiency, has been traditionally grouped with humoral immune deficiencies such as Common Variable Immune Deficiency (CVID) and X-linked agammaglobulinemia (XLA) despite the unique presence of the thymic neoplasm, late age of onset, frequent opportunistic infections and multiple cytopenias. Low prevalence and sporadic reporting have limited investigative efforts. We conducted a prospective, multimodal, study to establish the GS phenotype in the absence of severe infections. A previously characterized cohort of GS patients was compared to healthy individuals and those diagnosed with CVID or XLA using serology, immunophenotyping and histopathology. We also attempted to establish an inborn cause of the immune deficiency with HLA typing and Whole Exome Sequencing. GS patients exhibited severe hypogammaglobulinemia, a complete absence of B-cells in the peripheral blood and in biopsied primary and secondary lymphoid tissues. Total lymphocytes and CD4 T-cell counts were markedly reduced in GS but with intact T-cell proliferative capacity to in vitro mitogen and lectin stimulation. Autoantibody profiling identified high titers of antibodies in GS patient plasma to several cytokines, notably anti-interferons, which persisted for several years. These antibodies overlapped with those observed in thymoma patients without GS. We demonstrate that GS involves profound B-cell-loss, extending beyond blood to the lymphoid tissue, however plasma cells and circulating anti-cytokine antibodies can persist for years. In the absence of a unifying monogenic defect, the findings are suggestive of a thymoma-associated breakdown in central tolerance driving the immune dysregulation and deficiency seen in GS.

**Supplementary Information:**

The online version contains supplementary material available at 10.1007/s10875-026-01992-5.

## Introduction

Good’s syndrome (GS) lacks formal diagnostic criteria and an identifiable cause. Described by R.A. Good as an ‘association of thymoma and adult-onset immune deficiency’, patients present mostly with recurrent bacterial infections, hypogammaglobulinemia, and opportunistic infections (OIs) [1–4]. Initially considered a predominantly humoral immune deficiency, it was reclassified as a combined immune deficiency, and GS is now considered as a “phenocopy of inborn errors of immunity” [5–7]. Proposed mechanisms for disease pathogenesis include inborn genetic defects, epigenetic abnormalities, and dysregulated cytokine signalling affecting B cell development or thymic epithelial cell proliferation [1, 3, 8, 9]. The most recent update by the International Union of Immunological Societies (IUIS) states that GS has been associated with anti-cytokine autoantibodies [6, 10]. To our knowledge, these antibodies have never been systematically characterized in the clinical context and natural history of GS [11]. Considering that B cell lymphopenia and hypogammaglobulinemia are central characteristics of GS, understanding the origin, production and functional significance of anti-cytokine antibodies is essential. There have been few studies characterizing the baseline immune phenotype of GS, when patients are clinically stable, with comparisons to patients with related immune disorders and healthy controls [12, 13]. The extreme rarity of GS (estimated prevalence 1 per 700,000), compounded by diagnostic delays and frequent misclassification, has limited investigations and therefore its cause remains unknown [5].

In our previous single-center, multi-decade prospective study, we followed eight GS patients and characterized their long-term clinical progression and outcomes [14]. We documented the natural history of their disease: all patients had an unremarkable childhood, adolescence and early adulthood, but developed recurrent bacterial infections in the fourth or fifth decade of life, as well as severe and systemic OIs, making this syndrome a combined immune deficiency. In addition to the presence of a thymoma, we identified a complete absence of peripheral B-lymphocytes, and a multidecade progressive decline in lymphocytes, platelets, hemoglobin, and red blood cells, suggesting gradual bone marrow failure. This extensively characterized cohort with robust clinical, laboratory, tissue and genetic data, provided a unique opportunity to investigate the pathophysiology of GS.

We therefore conducted a study to compare GS patients to healthy individuals and other adults with humoral immune deficiencies (Common Variable Immune Deficiency, CVID, and X-linked agammaglobulinemia, XLA). All patients recruited in the study had been followed for years at the same specialized adult immune deficiency center. To assess the humoral and cellular components of all patients with immune deficiency, we performed blood and tissue phenotyping, as well as in vitro functional assays. Building on the recent recognition of anti-cytokine antibodies associated with infectious phenotypes, we also systematically characterized their presence, specificity and persistence in all our groups [15–17]. To identify a potential inborn error of immunity underlying GS, hitherto unknown, we performed Human leukocyte antigen (HLA) typing and whole-exome sequencing (WES). By studying GS in the context of these established immune deficiencies, we aimed to gain deeper insights into its underlying etiology and refine long-term patient management strategies.

## Methods

### Study Design

The study was approved by the McGill University Health Center Research Ethics Boards (MUHC-REB). All participants were over the age of 18 and provided informed written consent.

At study entry, all were stable clinically, without active infections or hospitalizations in the previous 3 months.

Patients with a diagnosis of either GS, CVID or XLA were recruited from the Immune Deficiency Treatment Center of the MUHC where they are followed prospectively every 6 months. Patients were diagnosed according to the European Society for Immunodeficiencies (ESID) diagnostic criteria, and for GS patients, based on our published criteria [14]. Healthy controls (HC) were recruited from a pool of individuals where immune deficiency and malignancies were ruled out. Full inclusion and exclusion criteria are provided in Supplementary Materials.

### Study Procedures

From each participant, a single blood draw of 45 ml was collected in tubes containing Ethylenediaminetetraacetic acid (EDTA, BD Biosciences), for plasma collection and flow cytometry, or acid citrate dextrose (ACD) for lymphocyte proliferation assays. At the time of collection, complete blood count (CBC) and serum immunoglobulin quantification were performed by the MUHC central laboratory. For individuals with a diagnosis of hypogammaglobulinemia, IgG values prior to immunoglobulin replacement therapy (IgRT) were extracted from their past medical records.

### Flow Cytometry

200 ul of whole blood in EDTA were incubated for 15 min at room temperature with a cocktail of monoclonal antibodies that included: Pacific Blue-CD3 (Clone UCHT1), BUV737-CD4 (SK3), APC H7-CD8 (SK1), PE-CF594-CD197 (CCR7, 150503) (BD Biosciences), BV510-CD19 (HIB19), PE-Cy7-CD45RA (HI100), BV650-CD16 (3G8), BV650-CD56 (5.1H11) (Biolegend) PerCP-Cy5.5-CD45 (2D1), Indo-1-Live Blue (Life Technologies). Following surface staining, FACS lysing solution (BD) was used to lyse red blood cells. All samples were analysed on a BD LSR Fortessa X-20 (BD Biosciences) and data from 100,000 lymphocytes were acquired. Data analysis was performed using FlowJo V10 (BD Biosciences).

### Lymphocyte Proliferation Assays

Peripheral blood mononuclear cells (PBMC) were purified from ACD blood using a Ficoll-Hypaque density gradient centrifugation. Freshly isolated lymphocytes were labelled with CFDA SE Tracer dye (Life Technologies) and either left unstimulated or stimulated with different lectins, mitogens and antigens: Phytohemagglutinin(PHA, Sigma), CD3-CLB and CD28-CLB(CD3-CD28, Cederlane Laboratories), Pokeweed(Sigma), Candida albicans (Stallergenes Greer), Tetanus toxoid(Sigma), cytomegalovirus antigen(CMV, Microbix), PepTivator® SARS-CoV-2 Prot_S Complete(Miltenyi Biotech inc) and inactivated influenza virus strains (FluLaval Tetra Vaccine). After a 7-day incubation period, the cells were washed and stained with the following antibodies: Pacific Blue-CD3, BUV737-CD4, APC H7-CD8, (BD Biosciences) PerCP-Cy5.5-CD45, Indo-1-Live Blue (Life Technologies). The CFDA SE-labelled lymphocytes were detected by flow cytometry. Data from 20,000 lymphocytes were acquired on a BD LSR Fortessa X-20 and analyzed with FlowJo V10 (BD Biosciences).

### Tissue Histology

Original biopsy specimens for clinical purposes preserved as formalin-fixed paraffin-embedded (FFPE) tissue blocks were sectioned and stained for immunohistochemistry (IHC). Detection was performed using a tyramide signal amplification system. HRP-Red chromogen (Roche) was used for CD3, CD20, and PAX5, while CD138 was visualized with DAB (brown) chromogen. Sections were counterstained with hematoxylin. Healthy lymph node and bone marrow tissues were obtained from the MUHC Department of Pathology and processed identically for comparison. Histological slides were examined using a Leica brightfield microscope at 40 × magnification; representative digital images were captured at no magnification and 10 × magnification using Aperio ImageScope software (version 12.4.6.5003). Quantification of positively stained cells in representative regions of each tissue section was performed using QuPath (version 0.5.1) (Supplementary Figures).

### Anti-Cytokine Antibody Measurement

Sequentially collected cryopreserved plasma samples from GS patients enrolled in our previous prospective study were analyzed and compared to the single timepoint plasma samples obtained from the HC, CVID and XLA participants. Additional plasma samples from individuals diagnosed with either a thymoma (Thymoma) or Thymic Squamous Cell Carcinoma (TSCC) were obtained from the MUHC Thoracic biobank. The MILLIPLEX® Human Cytokine Autoantibody Expanded IgG Magnetic Bead Panel was used according to manufacturer instructions. The bead-based 15-plex kit allowed for the simultaneous detection of autoantibodies against: BAFF, G-CSF, IFNβ, IFNγ, IL-1α, IL-6, IL-8, IL-10, IL-12 (p40), IL-15, IL-17A, IL-17F, IL-18, IL-22, and TNFα. Data acquisition and analysis were performed using the Luminex® analyzer (MAGPIX®) and xMAP technology.

### Statistical Analysis

CD4 and CD8 T cell proliferation in response to each stimulation condition was background-corrected by subtracting the corresponding unstimulated value for each individual sample. All immune phenotype and proliferation parameters were compared between the groups using the Kruskal Wallis test. Pairwise multiple comparisons between the independent groups utilized Dunn’s test. P values < 0.05 were considered significant. All significance tests were carried out using GraphPad Prism v8.

Threshold values for each anti-cytokine antibody were calculated as the median fluorescence intensity (MFI) of the healthy control (HC) group plus 2 standard deviations. To account for variation between parameters, MFI values for each sample were normalized by dividing by the respective threshold value. Normalized titer values for each antibody were visualized in a heatmap plot and color-coded in increasingly darker shades from white to red to signify the fold-changes over the threshold value.

### Genetic Screening

Genomic DNA was extracted from PBMCs of all GS patients using the QIAamp DNA Blood Mini Kit (QIAGEN) according to manufacturer’s instructions. High-resolution HLA typing was performed using NGSgo-MX-11 kit (GenDx) for multiplexed amplification of the following HLA-genes: HLA-A, HLA-B, HLA-C (whole gene), HLA-DRB1 (exon 2–3), HLA-DQB1 (exon 2–4), HLA-DPB1 (exon 2–5). Amplicons were sequenced on an Illumina platform and identified using the NGSengine GenDx software package.

### Whole Exome Sequencing (WES)

The gDNA quality was assessed on a TapeStation (Agilent), prior to library preparation (Lucigen). Exome capture was performed using the xGen Exome Research Panel v1.0 (IDT) and sequenced in a 100bp paired configuration on an Illumina NovaSeq 6000 instrument. WES data was analyzed for variants using GenPipes [PMID 31185495] version 4.3.0, following the BROAD Institute GATK best practices. Briefly, raw reads derived from the sequencing instrument were quality trimmed and adapter clipped using Trimmomatic to obtain a high-quality set of reads for sequence alignment (sam/bam) file generation [18]. The trimmed reads were aligned to the human reference genome assembly GRCh37 using a fast, memory-efficient Burrows-Wheeler transform (BWT) aligner BWA-MEM [19]. Mapped reads were refined using GATK and Picard program suites to improve mapping near insertions and deletions (indels; GATK indel realigner), remove duplicate reads with same paired start site (Picard mark duplicates) and improve quality scores (GATK base recalibration) [20, 21]. Variants were called using GATK haplotype caller in gVCF mode to allow efficient downstream merging of multiple samples into one variant file to streamline downstream variant processing procedures which include normalization and decomposition of multi-nucleotide polymorphisms, functional annotation with SnpEff and variant annotations using the Gemini framework which provides quality metric and extensive metadata to help further prioritize variants. Variants with sequence coverage below 20 reads were not considered [22–24].

## Results

### Demographics

**Fig. 1:**
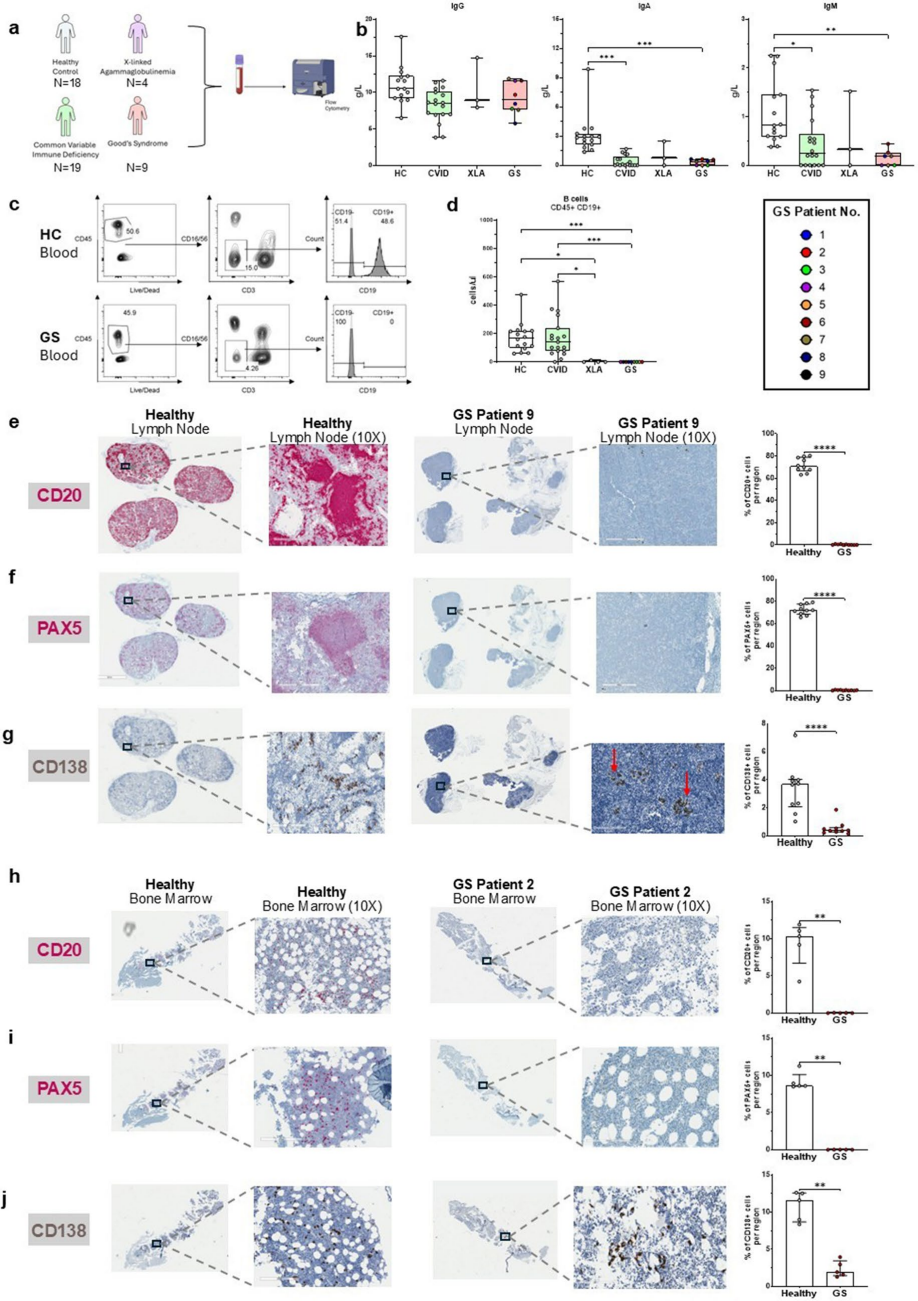
Humoral immune deficiency in Good’s Syndrome is characterized by pan-hypogammaglobulinemia and profound B cell deficiency in both blood and tissue. (**a**) Schematic representation of patient groups included in the cross-sectional immune phenotyping study. (**b**) Total serum IgG, IgA and IgM levels at recruitment across groups**.** (**c**) Representative flow cytometry dot plots showing total B lymphocytes (CD19 + CD45 + CD3-) in a healthy control (HC) (*top*) and GS patient (*bottom*). (**d**) Proportions and absolute counts of CD19 + B-cells in the blood from HC, CVID, XLA and GS patients (**e**–**g**) Immunohistochemistry (IHC) with hematoxylin counterstain of lymph node biopsies from a healthy individual and GS Patient 9, with representative 10 × magnified regions and quantification of positive cells across 10 regions of each tissue section (**e**) CD20 (HRP-red) (**f**) PAX5 (HRP-red) (**g**) CD138 (DAB). (**h**–**j**) IHC of bone marrow biopsies from a healthy control and GS Patient 2, with representative 10 × magnified regions and quantification across 5 regions of each tissue section with hematoxylin counterstain (**h**) CD20 (HRP-red) (**i**) PAX5 (HRP-red) (**j**) CD138 (DAB)

Thirty-two patients were recruited in this study: 9 patients with GS (of which 8 were previously prospectively characterized), 4 with XLA, 19 with CVID, in addition to 18 healthy controls (Fig. 1a). The mean age at the time of sample collection of GS and HC individuals (63.9 and 64.5 years old) was higher than XLA (49.5) and CVID individuals (53.9) (Table 1). All XLA individuals were males, while 77.8% of HC and GS groups, and 52.6% of CVID individuals were male.

**Table 1:** Group demographics for comparative immune phenotyping and in vitro T cell proliferation assessment

	Healthy controls (HC, *n* = 18)	Common variable immune deficiency (CVID, *n* = 19)	X-Linked Agammaglobulinemia (XLA, *n* = 4)	Good’s syndrome (GS, *n* = 9)
Age mean ± SD	67.8 ± 13.1	54.5 ± 13.2	49.6 ± 11.8	66.9 ± 14.0
Males N (%)	14 (77.8)	10 (52.6)	4 (100)	7 (77.8)
Race N (%)	White: 16 (88.8) Black: 2 (11.1)	White: 17 (89.4) East Asian: 2 (10.5)	White: 4 (100)	White: 8 (88.8) Black: 1 (11.1)
IgG prior to IgRT mean ± SD (g/L)	NA	3.49 ± 1.12	1.1	2.65 ± 2.35

At the time of study enrollment all XLA, CVID and GS individuals had been on IgG replacement (IgRT) for many years. Prior to the initiation of IgRT, serum IgG levels of all immune deficient individuals were very low; CVID individuals had a median of 3.49 g/L, while GS had 2.65 g/L and XLA had 1.1 g/L (Table 1).

### Assessment of Humoral Immunity

At the study visit, peripheral blood serum IgG levels were comparable between all groups. Serum IgM (median: 0.19g/L) and IgA (0.39g/L) were significantly lower in GS individuals compared to age and sex-matched HCs (0.82 and 2.85 g/L) (Fig. 1b). IgA was low in CVID (0.1 g/L) and XLA (0.74) individuals, whereas IgM levels varied across these groups. Flow cytometric phenotyping of peripheral lymphocytes revealed a complete absence of circulating B-cells (CD45 + CD19 +) in GS and XLA participants. B-cells in CVID individuals ranged from 0 to 22% of total CD45 + lymphocytes (0 to 569 cells/ul) (Fig. 1c).

Immunohistochemistry of an upper paratracheal lymph node from GS Patient 9 showed a near-complete absence of the B-cells with the CD20 and PAX5 stains (Fig. 1e and f). Similarly, B-cells were not detected in the bone marrow biopsy from GS Patient 2 (Fig. 1h and i). Plasma cells were detected in both GS biopsies of lymph node and bone marrow, with positive staining for CD138 (Fig. 1g and j). However, the CD138-positive plasma cells were reduced in both GS samples compared to the respective healthy tissues, where plasma cells were present in germinal centers and interfollicular regions.

### Assessment of Cellular Immunity

**Fig. 2:**
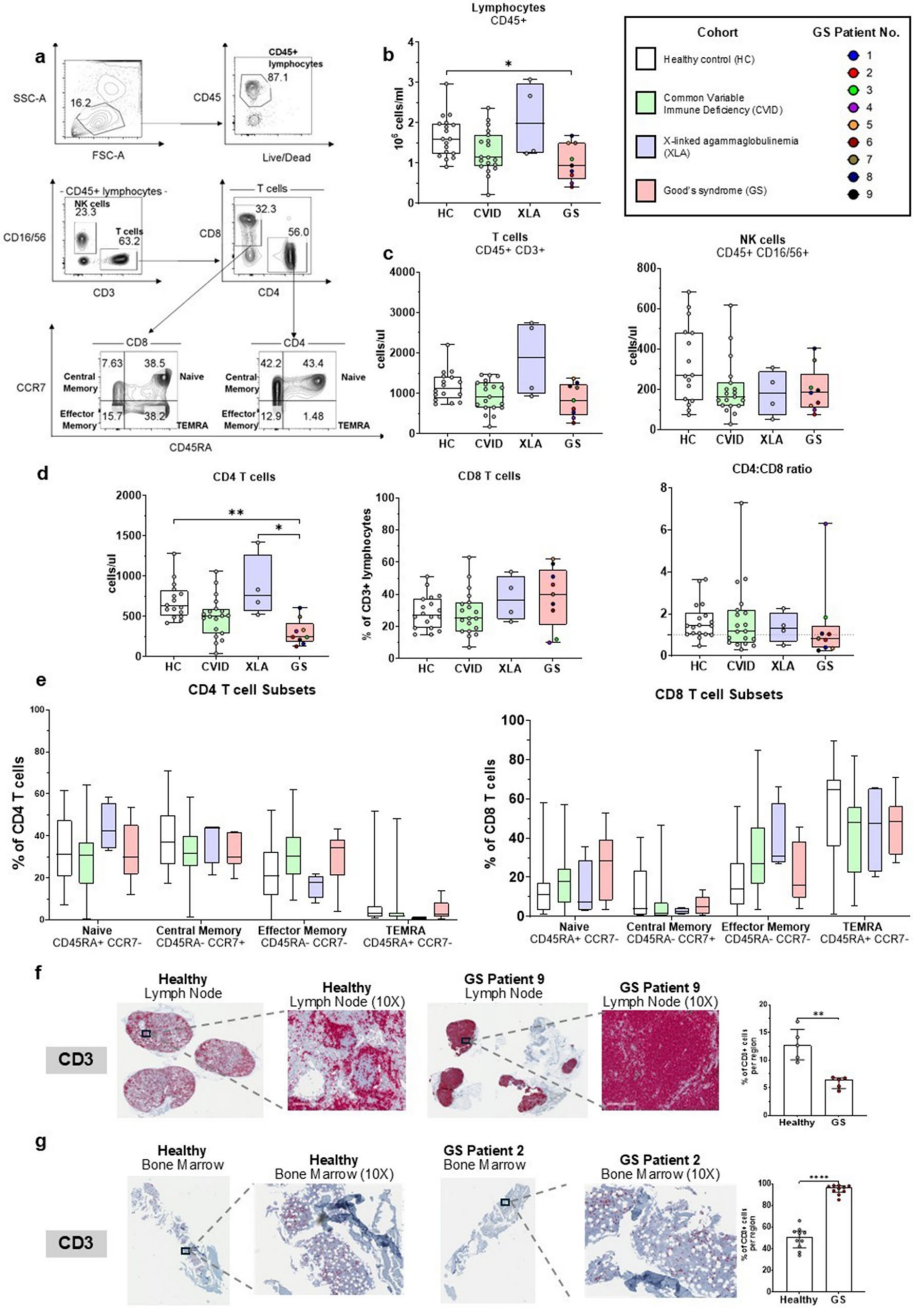
GS patients exhibit reduced peripheral lymphocytes with selective CD4 lymphopenia but T cell–dominated lymph node architecture. (**a**) Representative flow cytometric gating strategy for T and NK lymphocyte subsets from peripheral blood of a healthy control (HC). (**b**) Absolute counts of lymphocytes (CD45 +) (**c**) T cells (CD45 + CD3 +) and NK cells (CD45 + CD16/56 +) (**d**) CD4 and CD8 T cells and CD4:CD8 ratio in the blood of HC, CVID, XLA, and GS individuals. (**e**) Proportions of T cell memory subsets (Naïve, Central Memory, Effector Memory and TEMRA) of CD4 + and CD8 + T cells across all groups. (**f**) Immunohistochemistry (IHC) of CD3 (HRP-red) with hematoxylin counterstain of lymph node biopsies from a healthy individual and GS Patient 9, with representative 10 × magnified regions and quantification of positive cells across 10 regions of each tissue section (**g**) Immunohistochemistry (IHC) of CD3 (HRP-red) with hematoxylin counterstain of bone marrow biopsies from a healthy individual and GS Patient 9, with representative 10 × magnified regions and quantification of positive cells across 5 regions of each tissue section

GS patients had significantly lower total lymphocytes counts compared to HC (median: 0.94 *10^6^ vs 1.6 *10^6^ cells/ml) (Fig. 2b). There was no difference in total numbers of T-cells and NK-cells (Fig. 2c). The GS group had significantly lower numbers of CD4^+^ T cells (median: 245 *10^6^ cells/ml) compared to the HC (626) and XLA (754) groups, but no difference in CD8^+^ T-cell counts (Fig. 2d). No differences were observed between groups in the proportions of naïve and memory T cell subsets of CD4^+^ or CD8^+^ T cells (Fig. 2e). Both the lymph node and bone marrow biopsies of GS patients had positive staining for the CD3 T-cells. The lymph node of GS patient 9 consisted predominantly of CD3^+^ cells (Fig. 2f), contrasting with the healthy lymph node that had dense B-cell follicles surrounded by T cells. The GS bone marrow had a lower proportion of T cells compared to the healthy individual (Fig. 2g).

**Fig. 3:**
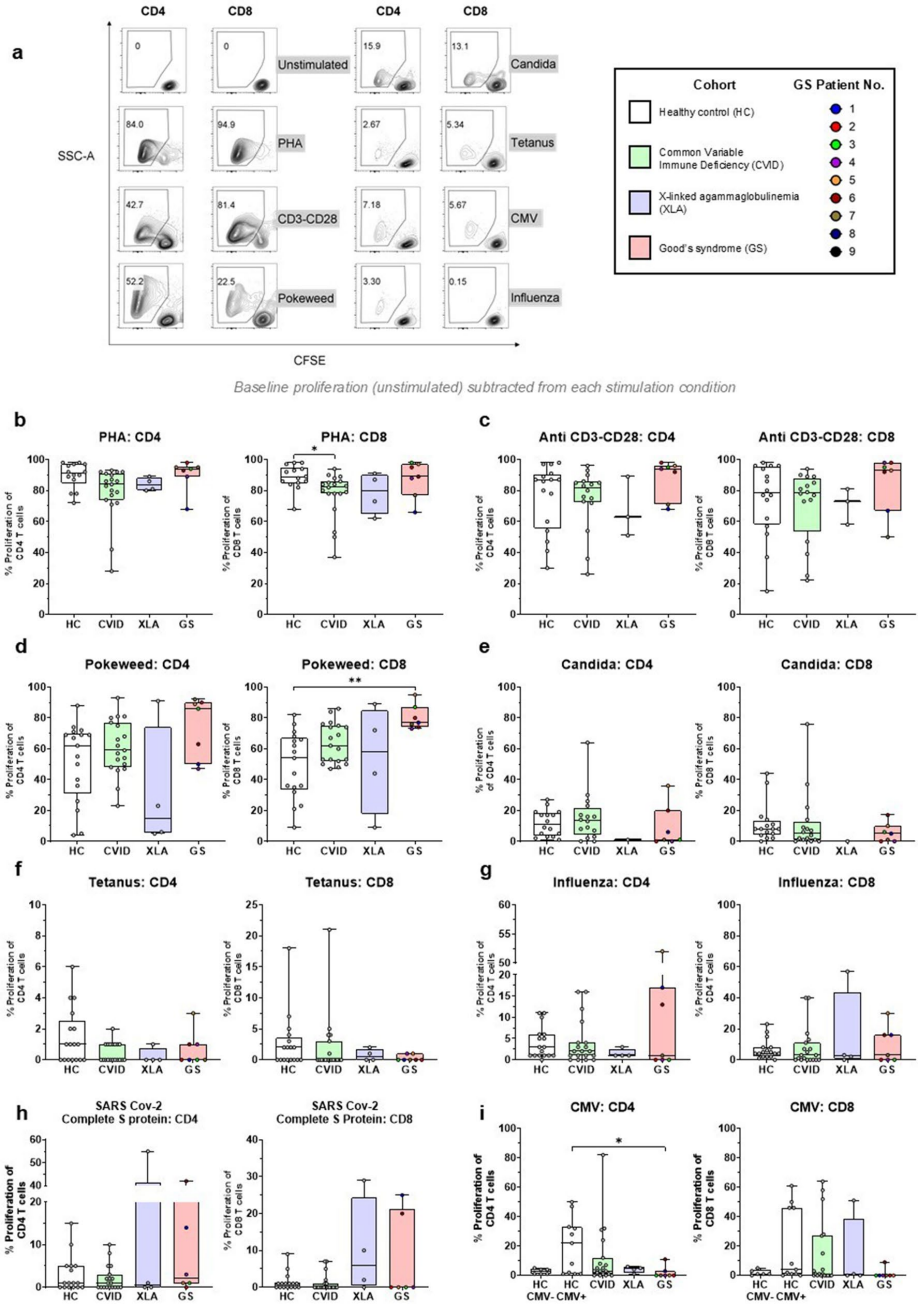
GS patient T cells retain proliferative capacity to mitogenic stimulation but reduced responses to CMV. (**a**) Schematic of the lymphocyte proliferation assay and representative flow cytometric plots showing proliferating CD4 and CD8 T cells from a HC following stimulation with mitogens or no stimulation (**b**-**i**) Proportions of proliferating CD4 and CD8 T cell across groups following whole lymphocyte stimulation with PHA, Anti-CD3-CD28, Pokeweed, Candida, Tetanus, Influenza, SARS-Cov-2 Complete S protein and CMV

T-cell proliferative capacity was assessed by measuring CD4^+^ and CD8^+^ proliferation following in vitro stimulation with different lectins, mitogens and antigens (Fig. 3). Proliferative response to non-specific stimulation (PHA, CD3-CD28, Pokeweed) of GS T cells was comparable to all other groups. In fact, GS patients had significantly higher proportions of proliferating CD8 T-cells upon pokeweed stimulation compared to HC (77% vs 54%, Fig. 3d). In response to PHA and CD3-CD28 stimulation, over 95% of both CD4^+^ and CD8^+^ T-cells of GS patients proliferated, in contrast to certain CVID individuals who had poor proliferative responses to all lectins and mitogens.

There was no significant proportional difference in CD4^+^ or CD8^+^ T cell proliferation between the HC, CVID, XLA or GS groups in response to specific antigenic stimulation such as Candida, tetanus, influenza or SARS-CoV-2 (Fig. 3e-h). The only significant difference was observed upon CMV peptide pool stimulation (Fig. 3i): CD4^+^ T-cells from GS patients (all seropositive for CMV) had lower proportional proliferation compared to the subset of HCs who were CMV seropositive (HC CMV +).

### Genetic Investigations

Whole exome sequencing (WES) was performed on 6 individuals with GS. Variants were annotated based on quality, allele frequency in public databases and for predicted functional impact. Different filtering approaches were considered to identify mutations or accumulating mutations in genes that could be associated with GS, including filtering to exclude common polymorphisms (allele frequency > 1% in gnomAD) and prioritization based on predicted deleteriousness (CADD score > 20). However, no shared or recurrent variants were identified nor any variants known to cause immune deficiencies. No candidate gene(s) met criteria for functional prioritization. High-resolution HLA typing revealed heterogeneous haplotypes among GS participants, with no shared allelic combination or enrichment of specific HLA class I or II alleles. We could not conduct statistical comparisons with a healthy population due to the small sample size and highly polymorphic HLA types (Table 2). These findings suggest that GS in our cohort is unlikely to be explained by a single shared monogenic defect or common HLA background, although heterogeneous rare variants or non-genetic mechanisms cannot be excluded.

**Table 2:** Results of HLA typing of 6 individuals with GS

Patient	1	2	3	4	5	6	7	8	9
HLA-A1	A*24:03	N/A	A*24:02	A*02:01	A*24:02	A*31:01	A*02:01	N/A	N/A
HLA-A2	A*68:01		A*26:01	A*03:01	A*25:01	A*33:03	A*03:02		
HLA-B1	B*07:05		B*18:01	B*07:02	B*35:01	B*15:16	B*51:01		
HLA-B2	B*18:01		B*45:01	B*44:02	B*35:08	B*53:01	B*58:01		
Bw	Bw6		Bw6	Bw6	Bw6	Bw4	Bw4		
Bw	Bw6		Bw6	Bw4	Bw6	Bw4	Bw4		
HLA-C1	C*12:03		C*06:02	C*05:01	C*04:01	C*04:01	C*03:02		
HLA-C2	C*15:05		C*07:01	C*07:02	C*12:03	C*14:02	C*15:02		
DRB1_1	DRB1*11:04		DRB1*01:01	DRB1*11:01	DRB1*04:02	DRB1*07:01	DRB1*11:04		
DRB1_2	DRB1*11:04		DRB1*15:01	DRB1*15:01	DRB1*15:02	DRB1*08:04	DRB1*13:03		
DQB1_1	DQB1*03:01		DQB1*05:01	DQB1*03:01	DQB1*03:02	DQB1*02:02	DQB1*03:01		
DQB1_2	DQB1*03:01		DQB1*06:02	DQB1*06:02	DQB1*06:01	DQB1*03:01	DQB1*03:01		
DPB1_1	DPB1*02:01		DPB1*04:01	DPB1*04:01	DPB1*02:01	DPB1*01:01	DPB1*04:02		
DPB1_2	DPB1*04:02		DPB1*04:01	DPB1*09:01	DPB1*04:01	DPB1*01:01	DPB1*23:01		

### Presence and Persistence of Anti-Cytokine Autoantibodies

**Fig. 4:**
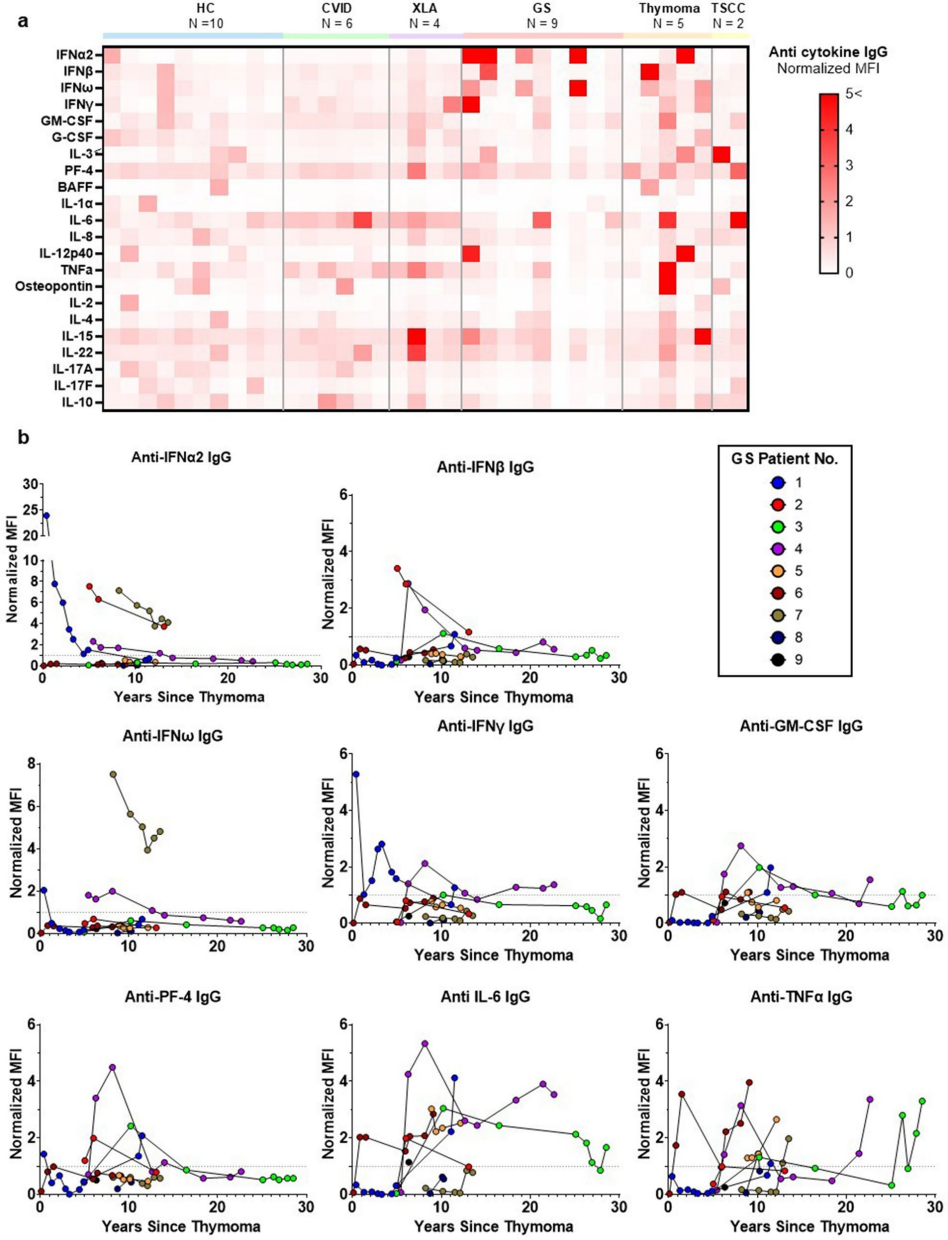
GS patients exhibit persistently high titers of thymoma-associated anti-cytokine autoantibodies. (**a**) Heatmap showing normalized median fluorescence intensity (MFI) of IgG autoantibodies targeting various cytokines (rows) across plasma samples (columns) from individuals with GS, XLA, CVID, HC, Thymoma, and Thymic Squamous Cell Carcinoma (TSCC). (**b**) Longitudinal levels for the selected autoantibodies (anti IFNα2, IFNβ, IFNω, IFNγ, GM-CSF, PF-4, IL-6, TNFα IgG) in individual GS patients in the years following thymectomy; the dotted line represents the positivity threshold (HC median + 2 SD)

To characterize thymoma-associated autoimmunity, anti-cytokine autoantibodies were measured in plasma of GS patients at multiple timepoints and compared with the other groups. At the earliest available post-thymectomy timepoint, several GS patients had high titers of anti-Type I interferons (α, β, ω) and Type II interferon (IFNγ) IgG (Fig. 4a). Additional antibodies targeting GM-CSF, PF-4, IL-6, IL-12p40, TNF-α, IL-15 and IL-22 were also detected in GS patients, and, in some cases, in the Thymoma and TSCC plasma obtained from the biobank. The highest titers were observed in GS Patient 1 against IFNα2 and IFNγ (24 and 5-folds, respectively, above the HC-derived threshold value), in the year following thymectomy.

Anti-interferons, GM-CSF and PF-4 antibodies were detected at multiple timepoints in GS patients, years apart, with titers consistently several folds higher than the threshold (Fig. 4b). No consistent trend was observed over time for a particular antibody across all GS patients, except a general decrease in anti-IFN-α2 and IFN-ω. Anti-TNFα IgG was found in all immune deficient groups and in one patient with thymoma. The levels fluctuated greatly over time in each GS patient.

## Discussion

Good’s Syndrome sets itself apart from other primary immune deficiencies by the presence of a thymic neoplasm, late age of onset, frequent opportunistic infections and multiple cytopenias. Yet, current treatment and clinical management follow the same approach as for the predominantly humoral deficiencies. This study addresses a critical knowledge gap by defining the serologic, cellular, and genetic features of GS in contrast to healthy and B-cell deficient counterparts. We confirmed a complete absence of B-cells not only in peripheral blood but also in tissues. In GS, the presence of plasma cells and residual, albeit low, levels of immunoglobulins, allude to the prior existence of antibody-producing cells and likely B-cell depletion in middle-age. GS patients, unlike the agammaglobulinemia group, had reduced CD4 T-cell counts but retained robust proliferative capacity to lectins and mitogens, even in individuals with OIs. We also detected high titer antibodies to multiple cytokines that persisted for years following the absence of B-cells. Genetic investigations revealed no shared pathogenic variants, consistent with the sporadic, adult-onset nature of the disease and pointing toward non-Mendelian or thymoma-associated mechanisms.

Pan-hypogammaglobulinemia and the complete lack of circulating B-cells in GS mirror the peripheral phenotype seen in XLA. In contrast, most CVID patients retained low to normal levels of circulating B cells. Tissue histopathology provided critical insights not previously confirmed in GS. First, the detection of CD138^+^ plasma cells in lymphoid tissues indicates that terminally differentiated B lineage cells are still present in tissue niches and that by extension, B-lymphocytes were once generated and could mature in GS patients. This could potentially account for the residual levels of serum immunoglobulins seen in patients and could be the source of the high titers of anti-cytokine antibodies observed in our cohort of GS patients. Second, staining for CD20 and PAX5 revealed that the absence of B-cells is not limited to the peripheral blood. CD20^+^ mature B cells were virtually absent in secondary lymphoid tissues, and PAX5^+^ cells, a marker of B lineage commitment (expressed from the pro-B cell stage), were severely reduced or absent in the bone marrow. This supports the idea that a potential defect in GS occurs at the level of early B-cell development, prior to egress from the bone marrow [25]. The alternate hypothesis (through unknown mechanisms) is that B-cells are subsequently depleted, not only in the blood but also in tissues. The lack of mature B-cells likely limits the generation and replenishment of mature plasma cells, which are present but reduced in GS, reflecting an eventual exhaustion of the B-lineage compartment.

Despite the clinical evidence of a combined immune deficiency (frequent OIs documented in our prior work), our immune phenotyping revealed that the cellular compartment was largely preserved [14]. Total T-cell and NK-cell counts were comparable to the healthy group, with the exception of a significant reduction in CD4^+^ T cells. This CD4 lymphopenia may be secondary to the absence of B cells and the disrupted lymphoid follicular architecture, both of which are essential to T cell activation and expansion [26–28]. However, this phenomenon was not observed in the XLA group that also lack B cells. Our immunoglobulin quantification and lymphocyte phenotyping results are largely consistent with laboratory findings reported in the literature and the few existing studies that directly compared GS to healthy or immunodeficient groups [4, 12, 13].

Importantly, we found no intrinsic defect in T-cell proliferative capacity, a finding that conventionally confirms in vitro cellular deficiency [29]. Thus, the mechanism underlying the documented susceptibility to OIs in our cohort remains unclear. We do acknowledge limitations in this experimental design. The lymphocyte proliferation assay (LPA) involved a 7-day culture which may have led to cell death, masking a survival bias towards proliferative cells. Moreover, we could not demonstrate normal antigen-specific T-cell recall responses, due to low responses across all groups and the lack of confirmed exposure/vaccination by history. Notably, we observed a lower GS CD4 proliferative response to CMV compared to healthy controls. However, this may be the result of CMV-driven exhaustion rather a primary T-cell defect, as several GS patients had past episodes of CMV disease or low level CMV viremia by PCR quantitative viral load measurements [30–33]. Further studies are needed to look at the complete spectrum of T cell responses from antigen recognition to the development of appropriate effector responses, including cytokine production and cytotoxicity.

Our WES analyses found no pathogenic genetic variants, shared across the cohort or in individual GS patients, that were both rare in the general population, and capable of explaining the severe immune deficiency or the specific block in B cell maturation. This was unsurprising given the sporadic and late-onset nature of GS. Our findings are limited by the small cohort size and the inability to perform genome-wide or HLA association studies to identify susceptibility alleles. Defects could be multigenic or may lie in the non-coding or promoter regions, not captured in the exome, but can affect age-related epigenetic changes.

Most notably, we found that GS patients had elevated IgG antibodies to multiple cytokines, including but not limited to anti-type I and II interferons. This study is the first systematic evaluation and documentation of anti-cytokine antibodies in a cohort of GS patients. The levels were contrasted with healthy individuals, those with humoral immune deficiencies, and patients with thymic neoplasms but no diagnosed immune deficiency. Despite all immunodeficient patients receiving immunoglobulin replacement therapy, the high titer anti-interferon autoantibodies were only noted in GS patients. Many of these anti-cytokine antibodies have been previously reported in thymoma patients or associated with infectious phenotypes (e.g., anti-type I IFNs and reduced JAK/STAT signalling, anti-IFN-γ and mycobacterial disease, anti-GM-CSF and cryptococcal meningitis), although their functional consequences remain unclear [11, 16, 34–36]. The anti-cytokine antibodies may not be pathogenic but may be secondary to severe infections and/or the resolution of inflammatory events. The presence of these antibodies in non-immune deficient thymoma patients, suggests that they are secondary to the thymic neoplasm and not specific to GS, challenging the idea that these autoantibodies underlie the immune deficiency phenotype seen in GS [17, 37]. Nevertheless, our findings support the notion that GS involves a broad spectrum of immune abnormalities, beyond those of hypogammaglobulinemia.

Although conceptually these antibodies could contribute to the decreased cellular immunity, it is unclear how they lead to the profound B-cell loss characteristic of GS. A possible explanation is that they could perturb the bone marrow microenvironment where cytokines such as IL-7, IL-6 and GM-CSF play important roles in hematopoiesis and particularly B lymphopoiesis [38–40]. Therefore, such factors may also contribute to the progressive cytopenias and bone marrow dysplasia we observed in the cohort [5]. Another possibility is the presence of pathogenic antibodies targeting B cells and not captured in our pre-determined panel. Such antibodies could mediate B-cell depletion through antibody-dependent cellular cytotoxicity (ADCC) or complement activation (CDCC). A relevant parallel exists in idiopathic CD4 lymphopenia: a recent study has described the existence and prevalence of pathogenic autoantibodies against CD4 T cells, which were capable of mediating complement- and antibody-dependant lysis of CD4 T cells [41]. By analogy, we can hypothesize that B cells were once present and following the incidence of the thymoma, a similar mechanism led to their depletion in the peripheral blood and tissues, resulting in the severe immunodeficiency phenotype and also notably, the CD4 lymphopenia noted in our GS cohort [42].

The human thymus plays a critical role in T cell maturation and deletion of autoreactive T cell clones through its autoimmune regulator (AIRE) protein-driven expression of tissue-specific antigens, [27, 43]. The development of a thymoma at an age when the thymus is typically involuted may have triggered a broader breakdown in central tolerance and allowed the escape of autoreactive T cells, potentially driven by reduced AIRE expression, a phenomenon increasingly recognized in Type I autoimmune polyendocrinopathy syndrome, myasthenia gravis (MG) and thymic epithelial tumors (TETs) [11, 44–46]. In this proposed model, T cells may directly target B lymphocytes, particularly early B cell precursors in the bone marrow. Findings from the study by Masci et al. may support this possibility, as an oligoclonal population of CD8^+^ T-cells with a conserved CDR3 motif were found to be expanded in the bone marrow of GS patients [47]. Although we did not find germline causes of immune deficiency, future investigations can be re-focused on tissue-specific somatic mutations, particularly in the thymic tumor microenvironment. Collectively, our observations support the reinterpretation of GS as an acquired, adult-onset phenocopy of inborn errors of immunity, rather than a classical primary immunodeficiency.

## Supplementary Information

Below is the link to the electronic supplementary material.

Supplementary file1 (PDF 477 KB)

## Data Availability

All data supporting the findings of this study are available within the paper and its Supplementary Information. All reagents and materials were commercially sourced and are detailed in the Methods section. Whole exome sequencing of Good's Syndrome patients is not available due to privacy reasons. All requests for data, protocols and further inquiries should be directed to christos.tsoukas@mcgill.ca.
