# Supplementary material for: Good’s Syndrome Mirrors a Combined Immunodeficiency with Anti-Cytokine Antibodies in the Total Absence of B Cells
Source: J Clin Immunol. 2026 Feb 17;46(1):26. doi: 10.1007/s10875-026-01992-5 (PMC12967467; doi:10.1007/s10875-026-01992-5)
Supplement: Supplementary file 1 — Supplementary file1 (PDF 477 KB) [file 10875_2026_1992_MOESM1_ESM.pdf]

Supplementary Materials

Criteria for study population recruitment

Inclusion Criteria:

All participants were over the age of 18 and at study entry were in a stable clinical state with no active infections or hospitalization in the previous 3 months.
Participants were grouped according to the following European Society for Immune Deficiency (ESID) diagnostic criteria, and for GS patients based on our published criteria [11].

X-linked Agammaglobulinemia (XLA):

Male patients with less than 2% CD19+ B cells and at least one of the following:

- Mutation in Btk
- Absent Btk mRNA on northern blot analysis of neutrophils or monocytes
- Absent Btk protein in monocytes or platelets
- Maternal cousins, uncles, or nephews with less than 2% CD19+ B cells

Common Variable Immune Deficiency (CVID):

Male or female patient who has a marked decrease of IgG (at least 2 SD below the mean for age) and a marked decrease in at least one of the isotypes IgM or IgA, and fulfills all of the following criteria:

- Onset of immunodeficiency at greater than 2 years of age
- Absent isohemagglutinins and/or poor response to vaccines upon challenge
- Exclusion of other causes of hypogammaglobulinemia

Good's syndrome (GS):

Male or female patient with a history of immune deficiency and thymoma (thymic neoplasm) with all of the following criteria:

- Onset of immunodeficiency at greater than 18 years of age
- Marked decrease of IgG (at least 2 SD below the mean for age) and a marked decrease in at least one of the isotypes IgM or IgA
- Absent isohemagglutinins and/or poor response to vaccines
- Defined causes of hypogammaglobulinemia have been excluded

Healthy Controls (HC):

Two healthy controls were recruited that were age and sex-matched to each GS patient. The healthy status was defined using our previously published criteria [12].

Exclusion Criteria:

Patients with secondary immunodeficiency with low or absent B cells secondary to chemotherapy, radiotherapy, biologic disease modulators and HIV were excluded.

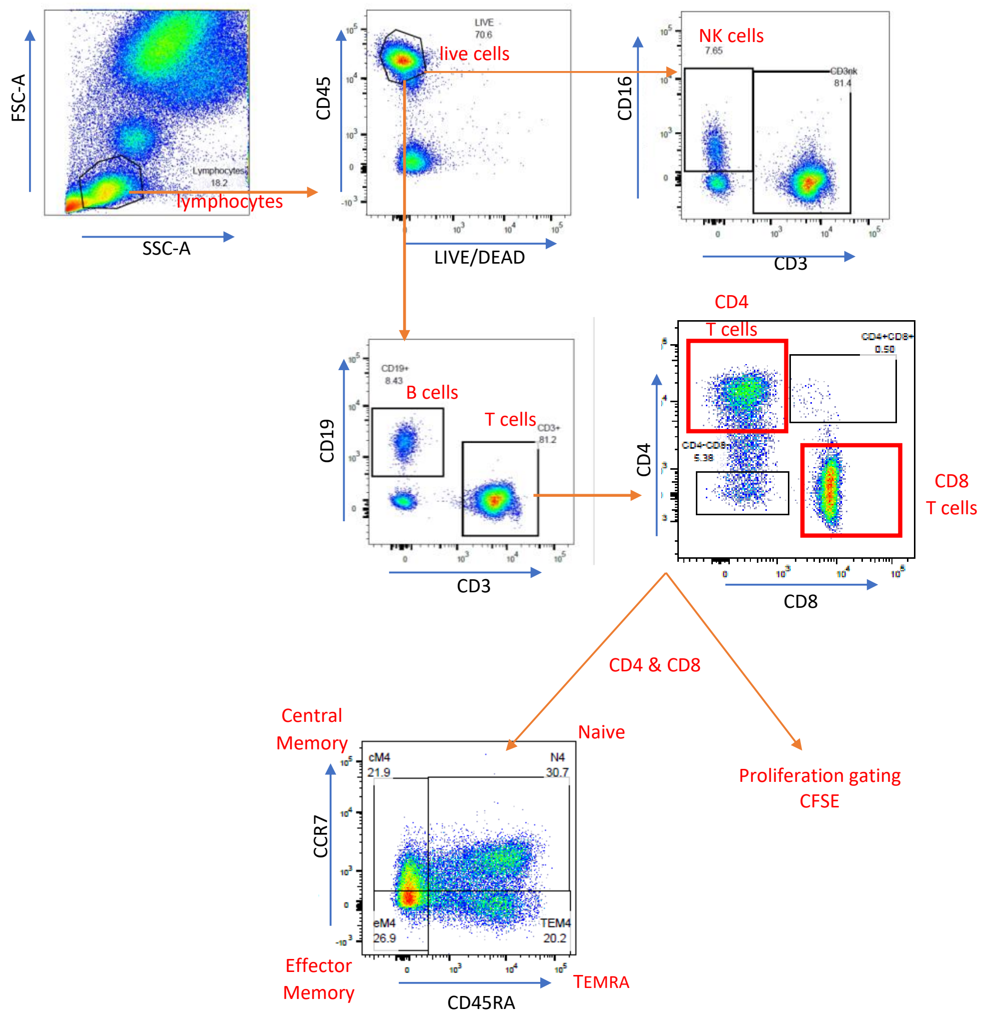

Supplementary Figure 1. Gating strategy for immune phenotyping of lymphocyte subsets performed on BD LSR Fortessa X-20

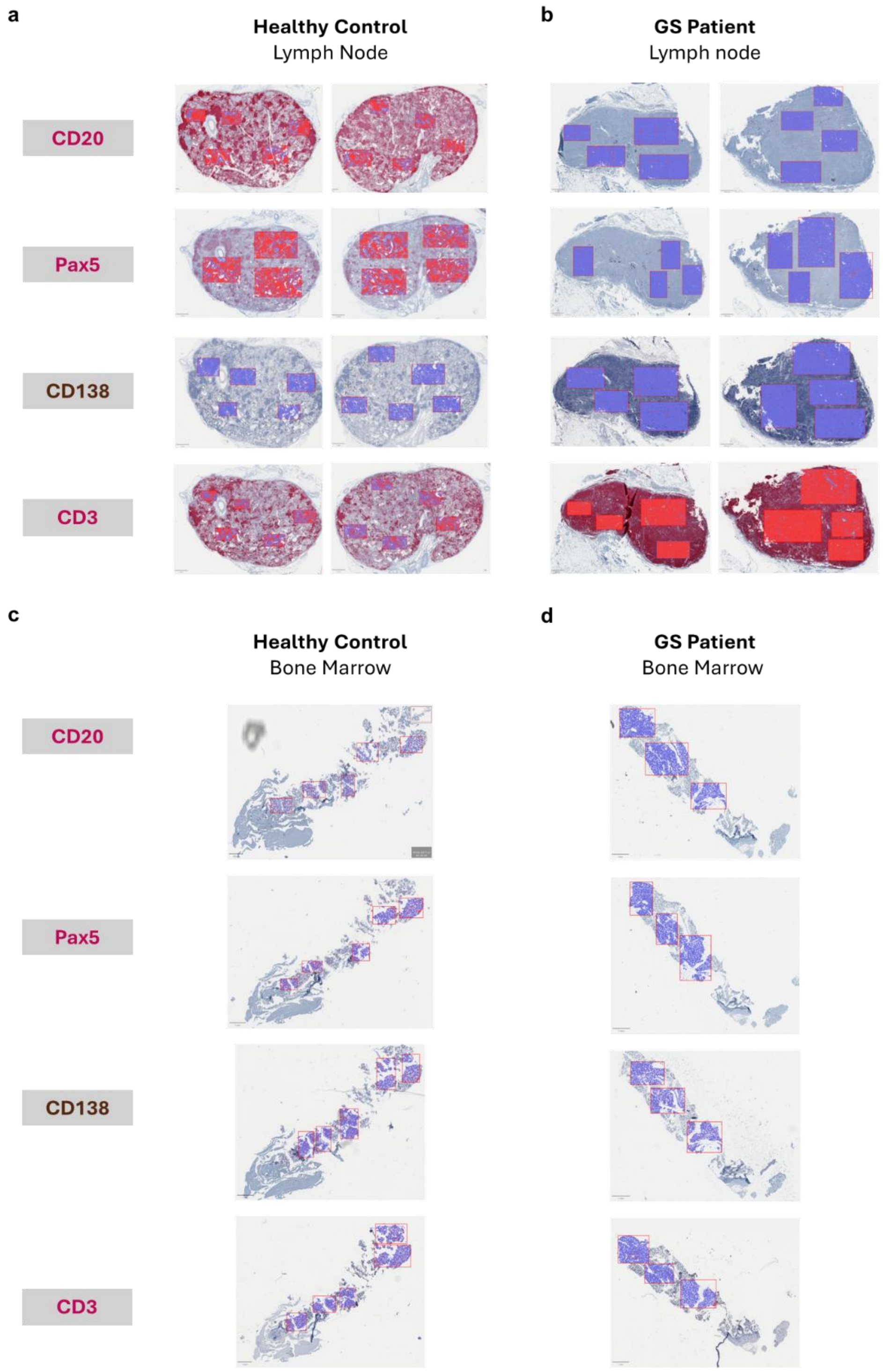

Supplementary Figure 2. Gating strategy for tissue immunohistochemistry quantification of marker expression using QuPath. Red outline boxes shown for representative regions used for positive cell quantification in **(a)** healthy lymph node, **(b)** GS lymph node, **(c)** healthy bone marrow, and **(d)** GS bone marrow sections. Within each outline region, positively stained cells are drawn in red by the program and negative cells in purple. Sections were stained for CD20, PAX5, CD3 (HRP-Red), and CD138 (DAB brown) and counterstained with hematoxylin.

Appendices

Participant	Sex	Years since diagnosis	IgG values prior to IgRT (g/L)	Age at immune phenotyping (yrs)	Age at autoantibody measurement (yrs)	CMV IgG serostatus	Ethnicity/ Race
**GS**							
1	M	2	1.09	61	58,59,60,69,70	Positive	Caucasian
2	M	13	4.00	70	62,63,70	Positive	Caucasian
3	M	25	2.40	83	62,68,74	Positive	Caucasian
4	F	18	<0.5	75	59,60,62	Positive	Caucasian
5	M	9	2.48	62	61,62,63	Positive	Caucasian
6	M	6	0.96	52	45,46,47	Positive	Black
7	F	9	6.3	44	41,43,44,45.46	Positive	Caucasian
8	M	2		87	86	Positive	Caucasian
9	M	1		68	68	Positive	Caucasian
**CVID**							
1	F	6.73	7	79		Positive	Caucasian
2	F	3.57	4	71			Caucasian
* 3	F	6.28	6	69			Caucasian
* 4	F	7.53	8	66		Positive	Caucasian
5	M	15.14	15	65		Positive	Caucasian
6	F	9.37	9	64		Positive	Caucasian
* 7	F	8.45	8	62		Positive	Caucasian
8	M	7.65	8	59		Positive	Caucasian
9	M	25.13	25	58		Positive	Caucasian
10	M	15.87	16	57		Positive	Caucasian
* 11	M	20.88	21	53		Positive	Caucasian
* 12	F	15.81	16	47		Positive	Caucasian
13	M	0.52	1	46		Negative	Caucasian
14	F	25.39	25	45		Positive	Caucasian
15	M	25.24	25	45		Positive	Caucasian
*16	M	12.19	12	44		Positive	Caucasian
17	M	57.80	58	42		Positive	Caucasian
18	F	14.48	14	37		Positive	Asian
19	M	15.54	16	27		Positive	Asian
**XLA**							
*1	M			64		Positive	Caucasian
*2	M		1.1	54		Positive	Caucasian
*3	M			42		Positive	Caucasian
*4	M			38		Positive	Caucasian
**HC**							
* 1	M	NA	NA	86		Positive	Caucasian
2	M	NA	NA	80		Negative	Caucasian
* 3	F	NA	NA	78		Positive	Caucasian
4	M	NA	NA	74		Positive	Caucasian
* 5	F	NA	NA	72			Caucasian
6	M	NA	NA	66		Positive	Caucasian
7	M	NA	NA	63		Negative	Caucasian
* 8	M	NA	NA	62		Positive	Caucasian
* 9	M	NA	NA	60		Negative	Caucasian
* 10	M	NA	NA	57		Positive	Black

*11	F	NA	NA	46		Positive	Caucasian
* 12	F	NA	NA	43		Negative	Caucasian
*13	M	NA	NA	64		Negative	Caucasian
14	M	NA	NA	52		Positive	Black
15	M	NA	NA	67		Positive	Caucasian
16	M	NA	NA	75			Caucasian
17	M	NA	NA	84		Positive	Caucasian
18	M	NA	NA	87		Positive	Caucasian

Supplementary Table 1. Demographics and clinical characteristics of all enrolled participants as per diagnosis. **GS:** Good's Syndrome, **CVID:** Common variable immune deficiency, **XLA:** X-linked agammaglobulinemia, **M**: male, **F**: female. **CMV:** cytomegalovirus. Minimum level of detection: IgG>0.5g/ml.
***:** Control individuals used for autoantibody measurement
